# Remote Heart Failure Patients Telemonitoring: Results of the TreC Heart Failure Study

**DOI:** 10.3390/jcdd12050182

**Published:** 2025-05-13

**Authors:** Massimiliano Maines, Annachiara Benini, Annalisa Vinci, Anna Manica, Elisa Erbogasto, Giancarlo Tomasi, Luisa Poian, Luigi Martinelli, Lorenzo Gios, Stefano Forti, Luigi Patil, William Mantovani, Maurizio Del Greco

**Affiliations:** 1 Division of Cardiology, Santa Maria del Carmine Hospital—Rovereto, Azienda Provinciale per i Servizi Sanitari (APSS), 38123 Trento, Italy; massimiliano.maines@apss.tn.it (M.M.); annalisa.vinci@apss.tn.it (A.V.); anna.manica@apss.tn.it (A.M.); giancarlo.tomasi@apss.tn.it (G.T.); luisa.poian@apss.tn.it (L.P.); maurizio.delgreco@apss.tn.it (M.D.G.); 2 Division of Cardiology, Verona University Hospital, 37129 Verona, Italy; elisa.erbo@gmail.com; 3 Division of Epidemiology, Azienda Provinciale per i Servizi Sanitari (APSS), 38123 Trento, Italy; luigi.martinelli@apss.tn.it (L.M.); william.mantovani@apss.tn.it (W.M.); 4 Bruno Kessler Foundation, 38123 Trento, Italy; lgios@fbk.eu (L.G.); forti@fbk.eu (S.F.); 5 Technology Department, Azienda Provinciale per i Servizi Sanitari (APSS), 38123 Trento, Italy; luigi.patil@apss.tn.it

**Keywords:** heart failure, telemonitoring, heart failure hospitalization

## Abstract

(1) **Aims:** In our study, we evaluated the effectiveness of a telemonitoring program based on a nursing clinic, supported by a physician who remotely monitors patients via a dedicated application (TreC Cardiology), in reducing visits and hospitalizations for HF in patients affected by HF living in Trentino in Italy. (2) **Methods and Results:** The TreC Heart Failure (TreC HF) study prospectively enrolled consecutive patients diagnosed with HF who attended our outpatient clinic and who were provided with the TreC Cardiology application. We analyzed primarily the number of visits and hospitalizations, comparing the year before and after the enrollment. From March 2021 to June 2023, we enrolled 211 patients, predominantly male (70.1%) and with a mean age of 71.5 ± 12.6 years. At baseline, 43.6% of patients were diagnosed with HFrEF, 28% with HFmrEF, and 28.4% with HFpEF. The mean left-ventricular ejection fraction (LV-EF) was 43.2 ± 11.9%. Outpatient visits in the year before the enrollment were on average 2.0 ± 1.2 vs. 1.6 ± 1.3 (*p* = 0.002) in the same following period. The percentage of patients who were hospitalized for heart failure went from 25.6% to 4.7% (*p* < 0.001). Analyzing HF categories separately, we found that, in the HFrEF population, after the enrollment, hospitalization for HF significantly decreased (32.6% vs. 7.6%, *p* < 0.001), while the number of outpatient visits did not vary (2.1 ± 1.4 vs. 2.1 ± 1.3, *p* = 0.795). In HFmrEF patients, both hospitalization for HF and outpatient visits significantly decreased (respectively, 30.5% vs. 1.7%, *p* < 0.001 and 2.0 ± 1.0 vs. 1.5 ± 1.3, *p* = 0.025). Finally, in the HFpEF population, only the number of outpatient visits significantly decreased after the enrollment (2.0 ± 1.1 vs. 1.0 ± 0.8, *p* < 0.001). (3) **Conclusions:** Our results confirm the enormous potential of telemonitoring, since in a real-world population affected by heart failure, it resulted in a significant reduction in hospitalization for HF and the number of outpatient visits.

## 1. Introduction

Heart failure (HF) is a complex clinical syndrome associated with a consistent risk of acute episodes of decompensation which can cause prolonged hospitalizations and can increase mortality and hospitalization recurrence [1,2,3,4,5]. Its prevalence appears to impact 1–2% of adults [6,7,8,9]. Therefore, efforts should be made to reduce its great burden of increased morbidity, mortality, and healthcare costs and to improve patients’ quality of life.

In the last few years, telemonitoring has emerged as a promising tool to reduce HF morbidity, HF mortality, and the HF-associated healthcare system burden [1,10]. In this context, HF symptoms and patients’ weight and parameters can be easily collected and remotely transmitted to optimize follow-up schedules and access to medical care. The recent COVID-19 pandemic highlighted some of the potential advantages of telemonitoring [11], especially when non-urgent visits were forcefully interrupted in many countries. However, different randomized controlled trials and observational studies led to conflicting results regarding the benefit of telemonitoring in HF [12,13,14,15]. Therefore, the 2021 European Society of Cardiology (ESC) guidelines suggested a weak (class IIb, level of evidence B) recommendation for telemonitoring in HF [1]. A possible explanation for these discrepancies could be the difference between monitoring methods [16] and model organization. Moreover, patients with unstable HF and a recent HF hospitalization could also modify the results, being at greater risk of a recurrent event [16]. Finally, as for other aspects of HF management, patients’ adherence may be incomplete. In any case, the interest in telemonitoring remains high, as it could be extremely valuable, especially in certain categories of HF patients, such as high-risk patients [17].

Trentino is an autonomous province of Italy in the country’s far north and is a mountainous province, therefore being characterized by valleys and municipalities, which could be located very far from a hospital. In this unique context, the chance to monitor patients remotely via a dedicated application appears crucial in order to plan only the follow-ups of the most severe patients in our clinic, while following stabilized patients remotely. This approach could also be environmentally friendly, sparing the travel to the hospital for many patients.

Thus far, no study has analyzed the impact of telemonitoring in a similar geographic context. Moreover, it appears crucial to identify which categories of patients most benefit from telemonitoring in order to address these efforts appropriately.

### Aims

The primary outcome of our study is to evaluate the effectiveness of a telemonitoring program based on a nursing clinic, supported by a physician who remotely monitors patients via a dedicated application (TreC Cardiology), in reducing visits and hospitalization for HF in patients affected by HF living in Trentino. Consequently, we performed the same analysis separately for patients affected by heart failure with reduced ejection fraction (HFrEF), heart failure with mildly reduced ejection fraction (HFmrEF), and heart failure with preserved ejection fraction (HFpEF).

## 2. Methods

### 2.1. TreC Cardiology Application

The TreC Cardiology application is a telemonitoring solution for heart failure management, featuring a smartphone app for patient use and a web-based platform for healthcare professionals to access and analyze patient data. This innovative solution was developed in collaboration with TrentinoSalute4.0, recognized as the “Competence Center on Digital Health”. TrentinoSalute4.0 is directed by a Steering Committee and an Executive Committee, with governance shared among the Autonomous Province of Trento, the Provincial Health Services Agency, and the Bruno Kessler Foundation. This collaborative structure ensures that TreC Cardiology is aligned with regional health policies, integrates seamlessly with the health system, and leverages cutting-edge research to support heart failure management in a digital health framework. Patients enter the weight, heart rate, and blood pressure into the application, confirm the prescribed therapy, and have a chat to communicate with the reference nurse and to send the results of the blood examination or other exams performed outside the hospital. Patients’ data are remotely processed during working hours from Monday to Friday. At our center, two nurses trained in HF and telemonitoring screen incoming data, and if some patient shows clinically relevant data, they contact a heart failure specialist cardiologist in order to solve the problem, via urgent teleconsultation, which can result in diuretic dose adjustment, therapy modification, or urgent access to the hospital. The TreC Cardiology application is prescribed by a cardiologist for a 2-year renewable period. In order to work properly, patients should be able to use a smartphone, or, alternatively, a caregiver could insert patients’ data, and they must live in a place with good smartphone connection.

### 2.2. Study Population

The TreC Heart Failure study prospectively enrolled consecutive patients diagnosed with HF who attended our outpatient clinic from March 2021 to June 2023 and who were provided with the TreC Cardiology application.

The inclusion criteria included patients affected by HF according to the ESC guidelines’ definition [1], aged ≥ 18 years, and the provision of informed consent to use the TreC Cardiology application.

We collected the clinical data and blood examinations of our population at baseline, namely the start date of telemonitoring, and at follow-up after 12 months.

Moreover, we analyzed how many times they transmitted data and used the chat. Finally, we recorded the number of outpatient visits and hospitalization for HF the year before and after the enrollment.

All data were anonymized. The study conformed to the Declaration of Helsinki and to local regulations for prospective studies and data handling.

### 2.3. Statistical Analysis

Continuous variables are presented as mean (standard deviation) or median (first-third quartile) based on their distribution; categorical data are reported as number and percentage.

Generalized estimating equations (GEEs) were used to perform both unadjusted and adjusted analyses in order to assess separately the risk of hospital admission for HF and outpatient visits, both before and after the introduction of the TreC Cardiology application.

GEE models with a binomial family and logit link were used to calculate ORs for hospital admissions due to HF (which was dichotomized) in a pre–post treatment comparison. IRRs were calculated using GEE models with a Poissonian family and log link in order to assess the impact of determinants on the risk of outpatient visits.

A *p*-value of *p* ≤ 0.05 was considered statistically significant.

All statistical analyses were conducted in Stata (16.1, StataCorp LLC, College Station, TX, USA).

## 3. Results

### 3.1. Study Population

We prospectively enrolled 211 patients, as summarized in Table 1, who were predominantly male (70.1%) and with a mean age of 71.5 ± 12.6 years. In our study population, 43.6% of patients were diagnosed with HFrEF, 28% with HFmrEF, and 28.4% with HFpEF. Analyzing the etiology of heart failure, 35.1% had dilated cardiomyopathy, 32.2% had ischemic heart disease, 15.2% had valvular heart disease, and 8.5% had hypertensive heart disease, with other etiologies accounting for 9% of patients. The mean left-ventricular ejection fraction (LV-EF) was 43.2 ± 11.9%. Cardiac magnetic resonance was performed in 71 patients, and late gadolinium enhancement (LGE) was found to be present in 53 of them (74.6%). Patients were predominantly in NYHA class II (56.9%). Atrial fibrillation was present in 42.2% of patients, while 26.1% of them had diabetes mellitus. Patients with a pacemaker made up 12.8%, those with an implantable cardioverter defibrillator (ICD) made up 38.9%, while concomitant cardiac resynchronization therapy (CRT) was present in 26.1% of the population. As concerning the TreC Cardiology application, 81% of patients regularly entered data into the application, with a data entry mean of 5.1 ± 6.8 in a month. Additionally, 51.7% of patients were able to use the application chat to communicate with our reference nurse. See Table 1 for other characteristics of the study population.

Follow-up visits were at a mean of 310.1 ± 198.3 days from the first visit. We compared the drug therapy of our population between baseline and follow-up, and it improved significantly in nearly all categories of heart failure drugs, while diuretic therapy significantly decreased (Table 2). We evaluated the percentage of patients affected by HFrEF in optimal medical therapy (OMT), defined as the presence of all four cornerstone drugs for HFrEF (beta-blockers; angiotensin-converting enzyme inhibitors, angiotensin receptor blockers, or angiotensin receptor–neprilysin inhibitors; mineralocorticoid receptor antagonists; and sodium–glucose cotransporter 2 inhibitors), and it increased from 34.4% at baseline to 55.1% at follow-up (*p* < 0.001).

### 3.2. Primary Outcome

**Figure 1:**
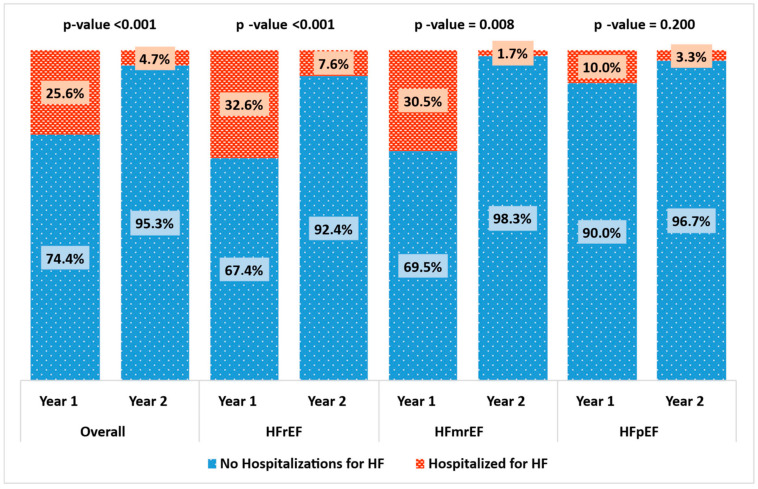
Hospitalization for HF before and after the introduction of the TreC Cardiology application.

**Figure 2:**
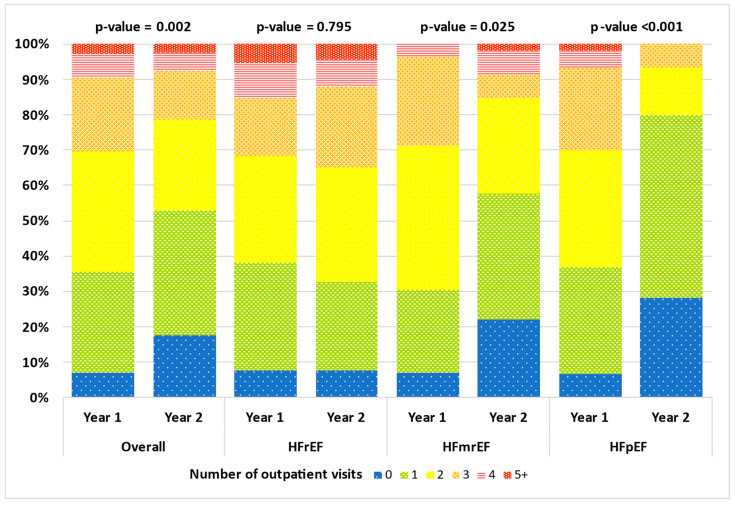
Outpatient visits before and after the introduction of the TreC Cardiology application.

Outpatient visits in the year before the enrollment were on average 2.0 ± 1.2 vs. 1.6 ± 1.3 (*p* = 0.002) in the same following period. The percentage of patients who were hospitalized for heart failure decreased from 25.6% to 4.7% (*p* < 0.001). Analyzing the HF categories separately, we found that in the HFrEF population after the enrollment, hospitalization for HF significantly decreased (32.6% vs. 7.6%, *p* < 0.001), while the number of outpatient visits did not vary (2.1 ± 1.4 vs. 2.1 ± 1.3, *p* = 0.795). In HFmrEF patients, both hospitalization for HF and outpatient visits significantly decreased (respectively, 30.5% vs. 1.7%, *p* < 0.001, and 2.0 ± 1.0 vs. 1.5 ± 1.3, *p* = 0.025). Finally, in the HFpEF population, only the number of outpatient visits significantly decreased after the enrollment (2.0 ± 1.1 vs. 1.0 ± 0.8, *p* < 0.001). See Table 3 for more information on primary outcomes and Table 4 for adjusted analysis of the risk of hospital admission for HF or outpatient visit before and after introduction of the TreC Cardiology application. This significant reduction in hospitalization for HF and in the number of outpatient visits are represented in Figure 1 and Figure 2, respectively.

Subsequently, we performed an adjusted multivariate analysis separately for the risk of hospital admission for HF and for the number of outpatient visits. Beside the primary outcome, we found that patients who had been hospitalized less for HF were those with less advanced NYHA class, higher LV-EF, with CRT, without atrial fibrillation, and non-diabetic patients. Regarding outpatient visits, they were significantly less frequent among patients with lower NYHA class, higher LV-EF, and without CRT. The utilization of the TreC Cardiology chat or more frequent monthly reports in the TreC Cardiology application were not associated with reduced hospitalization or visits. Also, the presence of a pacemaker was not significant. We did not include the variable OMT as covariate, since in the univariate analysis, it was not significantly associated with our outcomes. We therefore consider optimal medical therapy as an intermediate factor between exposure to telemedicine and the reduction in hospitalization risk, rather than as a confounder; telemedicine would facilitate therapy optimization, which in turn would reduce the risk of hospitalization for heart failure.

We performed a stratified adjusted analysis separately for HFrEF, HFmrEF, and HFpEF categories, which can be found in the Supplementary Materials.

## 4. Discussion

This real-world prospective study confirms the enormous potential of telemonitoring in significantly reducing hospitalization for HF and the number of follow-up visits.

Heart failure is responsible for 5–10% of all hospitalizations, and it is the most frequent cause of hospitalization in patients over 65 years of age [18,19]. Acute heart failure events are associated with an eight-fold increase in mortality and a nine-fold increase in the recurrence of hospitalization, and 75% of these hospitalizations occur in patients with a known diagnosis of heart failure [20]. Indeed, it is a pathology that has a significant impact on the quality of life and life expectancy of patients; moreover, it is related to high healthcare costs. In light of these data, it is mandatory to manage these patients by trying to prevent heart failure events. The latest guidelines on HF [1,2,21] report that an intensive strategy of the initiation and rapid up-titration of evidence-based treatment before discharge and during frequent and careful follow-up visits in the first 6 weeks following a HF hospitalization is recommended to reduce the risk of HF rehospitalization or death [22]. However, the resources to maintain these standards are limited, and waiting lists for clinical checks are often a problem. Indeed, in the last few years, telemonitoring has emerged as a promising tool to reduce HF morbidity, mortality, and the HF-associated healthcare system burden [1,10]. The 2021 European Society of Cardiology (ESC) guidelines suggested a class IIb, level of evidence B recommendation for telemonitoring in HF [1].

Since March 2021, we have provided the TreC Cardiology application to all patients diagnosed with heart failure who attended our division, and in light of this technological innovation, we have reorganized our heart failure clinic on the nurse case manager model. In our experience, we have chosen the model with a nurse case manager who remotely manages the patients, an electrophysiologist for arrhythmogenic or device problems, and a heart failure specialist to manage the clinical problems encountered during the remote monitoring of patients. This represents a paradigm shift in patient management. Given the limited resources, our strategy was to monitor all patients and space out visits for those with stable parameters regarding remote monitoring, thereby freeing up slots to focus on patients in the early discharge period or those who became unstable during follow-up, allowing us to see them within a few days in order to reassess their clinical condition and their therapy.

From the analysis of our data, the organizational change seems to be the independent factor that led to a gain in terms of the number of patients followed by the clinic, a reduction in follow-up visits, and, above all, a reduction in hospitalizations for heart failure after just one year of follow-up. Analyzing the different categories of the HF population separately, we observed that in the HFrEF population, the percentage of patients hospitalized for HF significantly reduced at follow-up, while the number of outpatient visits did not vary. We interpret this result considering that these patients are the most severe ones, and indeed, the physician is more likely to plan sooner follow-up visits. On the other hand, in the HFpEF population, the number of outpatient visits significantly decreased while we observed no change in hospital admissions for HF. However, the small data on hospitalization in HFpEF population could be result in bias in interpreting this result.

The implementation of the four disease-modifying drugs has changed the prognosis of HFrEF patients; indeed, we have also considered this aspect, showing that the percentage of HFrEF patients in optimal medical therapy (OMT), defined as the presence of all four cornerstone drugs for HFrEF (beta-blockers; angiotensin-converting enzyme inhibitors angiotensin receptor blockers or angiotensin receptor-neprilysin inhibitors; mineralocorticoid receptor antagonists; and sodium-glucose cotransporter 2 inhibitors), significantly increased from baseline to follow-up. Indeed, we performed a univariate analysis, which showed that the variable OMT was not significantly associated with our primary outcome, namely the reduction in HF hospitalization and follow-up visits. Of note, we also observed that patients who transmitted data more frequently had similar numbers of HF admission and follow-up visits, in accordance with the advantages of the TreC application, which extended to all patients, improving their drug therapy remotely and improving the response times to cardiological alerts.

Previous studies on telemonitoring have reported conflicting results. Since there is extreme heterogeneity in the different types of telemedicine and healthcare systems, it is fundamental to evaluate each approach individually and, over the long term, to understand their usefulness. Moreover, it is crucial to identify the category of patients which most benefits from a telemonitoring program, especially in the worldwide expansion of digital cardiology. This represents an additional advance in an HF approach tailored to the patient and their context. Our study confirms for the first time in this geographic alpine context the benefit of a telemonitoring program in reducing HF hospitalization and follow-up visits and, interestingly, separately analyzes these findings among HF categories.

### Strength and Limitations

This study on telemonitoring for heart failure highlights both significant strengths and some limitations, offering a valuable analysis of a real-world healthcare solution. The topic itself is especially timely and relevant, given the increased demand for remote healthcare options post-COVID-19. In particular, this study explores the potential of telemonitoring to address the high healthcare burdens associated with HF by reducing hospitalizations and outpatient visits.

However, some limitations deserve consideration. First of all, this is a before-and-after analysis without a comparator group, and this could introduce significant potential for bias. We are collecting data on a similar population of HF patients which did not receive the application, but data are not yet available. We are aware that this limitation gives partial credibility to the study, but we wanted to raise a first hypothesis in this context, which of course will be worthy of proper analysis. The 12-month follow-up period limits our understanding of long-term effects on health outcomes and healthcare costs. A longer follow-up could reveal whether the benefits of telemonitoring are sustained over time and how adherence rates might change. Furthermore, although the study adjusted for several variables, some confounders—such as socioeconomic status, education level, access to caregivers, and variations in patient adherence—were not fully accounted for, which could influence outcomes. The number of patients involved in telemonitoring was limited. Since we wanted to include all patients who first received the application in order to understand the usefulness of this new approach, especially in our geographic context, in our study, we included both high-risk patients in the most vulnerable early post-discharge phase and stable chronic HF patients, who theoretically represent two different categories of patients. Furthermore, 65% of our patients had ICDs or CRTs, and these devices have evolved over time from tools for the treatment of arrhythmias and the synchronization of cardiac contraction to devices that allow for the management of the pathology by allowing for the continuous monitoring of a series of parameters. In the TreC Cardiology application, electrophysiological alerts were not included. However, if we detected a clinical alert, our reference nurse was able to analyze remotely the parameters transmitted by the device in order to integrate these data together.

## 5. Conclusions

Our results suggest the enormous potential of telemonitoring, since, in a real-world population affected by heart failure, it resulted in a significant reduction in hospitalization for HF and the number of outpatient visits. Of course, randomized controlled trials (RCTs) and extended follow-up are needed in order to assess the long-term outcomes and sustainability of the intervention’s benefits.

## Figures and Tables

**Table 1:** Characteristics of the study population at baseline.

Study Population	
N	211
Age, years	71.5 ± 12.6
Male sex, %	70.1%
Etiology, %	
Ischemic heart disease	32.2%
Dilated cardiomyopathy	35.1%
Valvular heart disease	15.2%
Hypertensive heart disease	8.5%
Other	9.0%
Heart failure, %	
HFrEF	43.6%
HFmrEF	28.0%
HFpEF	28.4%
LV-EF, %	43.2 ± 11.9
LGE, %	74.6%
NYHA class, %	
I	28.9%
II	56.9%
III	13.7%
IV	0.5%
Atrial fibrillation, %	42.2%
Paroxysmal	17.5%
Persistent	7.6%
Permanent	17.5%
Diabetes mellitus, %	26.1%
Device, %	
Pacemaker	12.8%
ICD	38.9%
CRT	26.1%
Laboratory	
Hemoglobin, g/dL	13.5 ± 1.8
Creatinine, mg/dL,	1.2 ± 0.5
Potassium, mg/dL	4.5 ± 0.5
NT-proBNP, pg/mL *	1783 (530–4180)
TreC chat utilization, %	51.7%
TreC data entry, %	81.0%
N° data entry/month	5.1 ± 6.8

All values are reported as mean ± standard deviation or percentage. * NT-proBNP is reported as the median (first quartile–third quartile). CRT: cardiac resynchronization therapy; HFmrEF: heart failure with mildly reduced ejection fraction; HFpEF: heart failure with preserved ejection fraction; HFrEF: heart failure with reduced ejection fraction; ICD: implantable cardioverter defibrillator; LGE: late gadolinium enhancement; LV-EF: left-ventricular ejection fraction; NYHA: New York Heart Association.

**Table 2:** Differences between therapy at baseline and follow-up.

Therapy	Year Pre-TreC	Year Post-TreC	*p*-Value
BB	84.4%	88.0%	**0.026**
ACEi	39.8%	30.2%	**0.003**
ARB	13.7%	16.7%	0.088
ARNI	26.5%	35.9%	**<0.001**
MRA	64.9%	70.3%	**0.022**
SGLT2i	26.1%	35.9%	**0.001**
Furosemide	66.4%	59.9%	**0.009**
OMT in HFrEF	34.4%	55.1%	**<0.001**

ACEi: angiotensin-converting enzyme inhibitor; ARB: angiotensin receptor blocker; ARNI: angiotensin receptor–neprilysin inhibitor; BB: beta-blocker; HFrEF: heart failure with reduced ejection fraction; MRA: mineralocorticoid receptor antagonist; OMT: optimal medical therapy; SGLT2i: sodium-glucose cotransporter-2 inhibitor.

**Table 3:** Primary outcome: differences between outpatient visits (mean + SD) and hospitalization for HF (percentage of patients who were admitted for HF) in patients affected by HF before and after the introduction of the TreC Cardiology application.

	Outpatient Visits			Hospital Admission for HF		
	Year Pre-TreC	Year Post-TreC	*p*-Value	Year Pre-TreC	Year Post-TreC	*p*-Value
**Study population**	2.0 ± 1.2	1.6 ± 1.3	**0.002**	25.6%	4.7%	**<0.001**
**HFrEF population**	2.1 ± 1.4	2.1 ± 1.3	0.795	32.6%	7.6%	**<0.001**
**HFmrEF population**	2.0 ± 1.0	1.5 ± 1.3	**0.025**	30.5%	1.7%	**0.008**
**HFpEF population**	2.0 ± 1.1	1.0 ± 0.8	**<0.001**	10.0%	3.3%	0.200

HFmrEF: heart failure with mildly reduced ejection fraction; HFpEF: heart failure with preserved ejection fraction; HFrEF: heart failure with reduced ejection fraction.

**Table 4:** Adjusted analysis of the risk of hospital admission for HF or outpatient visits before and after the introduction of the TreC Cardiology application.

	HF Hospital Admissions OR (95% CI)	*p*-Value	Outpatient Visits IRR (95% CI)	*p*-Value
TreC introduction	0.08 (0.04–0.19)	**<0.001**	0.81 (0.71–0.92)	**0.002**
Age	1.01 (0.98–1.04)	0.623	0.99 (0.99–1.00)	**0.039**
Female sex	0.82 (0.35–1.93)	0.650	1.11 (0.07–1.27)	0.130
Monthly reports	0.98 (0.92–1.04)	0.424	1.01 (1.00–1.01)	0.217
TreC chat utilization	1.08 (0.52–2.23)	0.840	0.99 (0.87–1.12)	0.845
NYHA class	5.25 (3.06–8.99)	**<0.001**	1.12 (1.02–1.23)	**0.015**
LV-EF	0.95 (0.92–0.98)	**0.001**	0.99 (0.98–0.99)	**<0.001**
Atrial fibrillation	2.30 (1.16–4.58)	**0.017**	1.18 (1.04–1.33)	**0.011**
PM	1.25 (0.46–3.41)	0.659	1.01 (0.85–1.19)	0.950
CRT	0.40 (0.17–0.95)	**0.037**	1.16 (1.02–1.32)	**0.020**
Diabetes mellitus	2.31 (1.12–4.74)	**0.023**	1.01 (0.88–1.16)	0.884

Results are obtained using generalized estimating equation models. GEE models with binomial family and logit link were used to calculate OR for HF hospital admissions. GEE models with Poissonian family and log link were used to calculate IRR of outpatient visits. CRT: cardiac resynchronization therapy; PM: implantable pacemaker; LV-EF: left-ventricular ejection fraction; NYHA: New York Heart Association.

## Data Availability

The original contributions presented in this study are included in the article/Supplementary Materials. Further inquiries can be directed to the corresponding author.

## Supplementary Materials

The following supporting information can be downloaded at https://www.mdpi.com/article/10.3390/jcdd12050182/s1, Table S1: Adjusted analysis of the risk of hospital admission for HF, or outpatient visits, before and after the introduction of TreC Cardiology application in HFrEF population; Table S2: Adjusted analysis of the risk of hospital admission for HF, or outpatient visits, before and after the introduction of TreC Cardiology application in HFmrEF population; Table S3: Adjusted analysis of the risk of hospital admission for HF, or outpatient visits, before and after the introduction of TreC Cardiology application in HFpEF population.
