# Supplementary material for: Remote Heart Failure Patients Telemonitoring: Results of the TreC Heart Failure Study
Source: J Cardiovasc Dev Dis. 2025 May 13;12(5):182. doi: 10.3390/jcdd12050182 (PMC12111988; doi:10.3390/jcdd12050182)
Supplement: Supplementary file 1 [file jcdd-12-00182-s001.zip › jcdd-3572561-supplementary.pdf]

Table S1. Adjusted analysis of the risk of hospital admission for HF, or outpatient visits, before and after the introduction of TreC Cardiology application in HFrEF population.

	HF hospital admissions OR (95% CI)	p-value	Outpatient visits IRR (95% CI)	p-value
TreC introduction	0.07 (0.03-0.20)	**<0.001**	1.03 (0.84-1.25)	0.795
Age	1.01 (0.97-1.06)	0.540	0.99 (0.99-1.00)	0.168
Female sex	1.39 (0.28-6.75)	0.686	0.99 (0.77-1.27)	0.927
Monthly reports	0.96 (0.89-1.04)	0.331	1.00 (0.99-1.01)	0.927
TreC chat utilization	0.67 (0.22-2.04)	0.483	0.88 (0.74-1.06)	0.181
NYHA class	5.30 (2.45-11.47)	**<0.001**	1.15 (1.02-1.29)	**0.022**
EF	0.89 (0.80-0.99)	**0.038**	0.99 (0.98-1.01)	0.531
AF	2.79 (1.09-7.11)	**0.032**	1.14 (0.97-1.34)	0.107
PM	0.90 (0.04-18.49)	0.947	1.05 (0.76-1.44)	0.770
CRT	0.24 (0.07-0.90)	**0.034**	1.03 (0.85-1.26)	0.736
DM	5.76 (1.66-19.20)	**0.006**	1.07 (1.21-1.27)	0.432

Table S2. Adjusted analysis of the risk of hospital admission for HF, or outpatient visits, before and after the introduction of TreC Cardiology application in HFmrEF population.

	HF Hospital Admissions OR (95% CI)	p-value	Outpatient Visits IRR (95% CI)	p-value
TreC introduction	0.02 (0.00-0.35)	**0.008**	0.76 (0.59-0.97)	**0.025**
Age	1.03 (0.98-1.08)	0.250	1.00 (0.99-1.01)	0.804
Female sex	0.28 (0.05-1.70)	0.166	1.00 (0.77-1.30)	0.981
Monthly reports	0.99 (0.92-1.07)	0.869	1.01 (0.98-1.04)	0.432
TreC chat utilization	4.47 (0.85-23.39)	0.076	1.35 (1.02-1.78)	**0.034**
NYHA class	4.13 (1.57-10.85)	**0.004**	0.88 (0.72-1.09)	0.237
EF	1.11 (0.89-1.40)	0.336	1.01 (0.98-1.05)	0.431
AF	1.20 (0.34-4.17)	0.779	1.25 (0.92-1.70)	0.154
PM	3.59 (0.58-22.02)	0.168	1.37 (1.03-1.83)	**0.033**
CRT	0.30 (0.07-1.37)	0.121	1.21 (0.95-1.55)	0.125
DM	0.52 (0.13-2.02)	0.343	0.88 (0.65-1.19)	0.421

Table S3. Adjusted analysis of the risk of hospital admission for HF, or outpatient visits, before and after the introduction of TreC Cardiology application in HFpEF population.

	HF Hospital Admissions OR (95% CI)	p-value	Outpatient Visits IRR (95% CI)	p-value
TreC introduction	0.22 (0.02-2.23)	0.200	0.50 (0.39-0.64)	**<0.001**
Age	0.99 (0.94-1.04)	0.759	0.99 (0.98-1.00)	0.175
Female sex	0.57 (0.10-3.12)	0.513	1.32 (1.06-1.64)	**0.014**
Monthly reports	0.91 (0.63-1.31)	0.598	1.01 (1.00-1.026)	0.050
TreC chat utilization	0.63 (0.03-13.06)	0.768	0.86 (0.66-1.12)	0.272
NYHA class	23.75 (5.17-109.11)	**<0.001**	1.31 (1.04-1.65)	**0.022**
EF	0.96 (0.73-1.27)	0.798	1.01 (0.98-1.03)	0.613
AF	1.19 (0.16-8.62)	0.862	1.00 (0.74-1.35)	0.995
PM	2.06 (0.17-24.53)	0.566	0.89 (0.63-1.27)	0.532
CRT	1.64 (0.26-10.32)	0.600	1.65 (1.37-2.00)	**<0.001**
DM	0.15 (0.02-1.41)	0.098	0.94 (0.69-1.28)	0.693
